# 3-Bromo-5-meth­oxy-4-(4-methyl­piperidin-1-yl)furan-2(5*H*)-one

**DOI:** 10.1107/S1600536811008804

**Published:** 2011-03-12

**Authors:** Xin-Ping Wei, Jian-Hua Fu, Yue-He Tan, Zhao-Yang Wang

**Affiliations:** a School of Chemistry and Environment, South China Normal University, Guangzhou 510006, People’s Republic of China

## Abstract

There are two molecules in the asymmetric unit of title compound, C_11_H_16_BrNO_3_, which was obtained *via* the tandem Michael addition–elimination reaction of 3,4-dibromo-5-meth­oxy­furan-2(5*H*)-one and 4-methyl­piperidine in the presence of potassium fluoride. The furan­one rings are approximately planar [maximum atomic deviations of 0.026 (2) and 0.015 (2) Å, respectively]. The packing is stabilized by weak inter­molecular C—H⋯O and C—H⋯Br inter­actions.

## Related literature

For biologically active 4-amino-2(5*H*)-furan­ones, see: Lattmann *et al.* (1999 ▶, 2005 ▶, 2006 ▶). For natural and synthetic products of 2(5*H*)-furan­ones, see: Zhou *et al.* (2009 ▶). For the synthesis of the title compound, see: Song, Wang *et al.* (2009 ▶). For a related structure, see Song, Li *et al.* (2009 ▶).
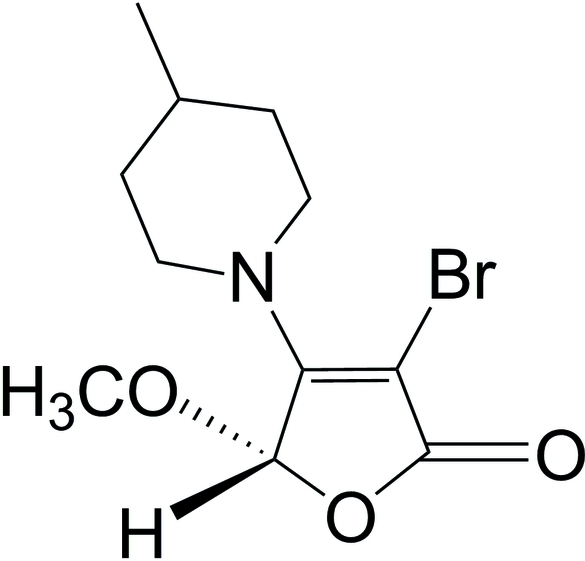


## Experimental

### 

#### Crystal data

C_11_H_16_BrNO_3_
*M* *_r_* = 290.15
Monoclinic, 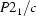

*a* = 12.681 (3) Å
*b* = 10.481 (2) Å
*c* = 19.947 (4) Å
β = 103.312 (3)°
*V* = 2579.9 (9) Å^3^
*Z* = 8
Mo *K*α radiation
μ = 3.18 mm^−1^
*T* = 298 K
0.32 × 0.22 × 0.20 mm

#### Data collection

Bruker APEXII area-detector diffractometer
Absorption correction: multi-scan (*SADABS*; Sheldrick, 1996 ▶) *T*_min_ = 0.436, *T*_max_ = 0.529
9692 measured reflections
4532 independent reflections
1918 reflections with *I* > 2σ(*I*)
*R*_int_ = 0.071

#### Refinement

*R*[*F* ^2^ > 2σ(*F* ^2^)] = 0.049
*wR*(*F* ^2^) = 0.109
*S* = 1.08
4532 reflections
294 parameters
H-atom parameters constrained
Δρ_max_ = 0.53 e Å^−3^
Δρ_min_ = −0.40 e Å^−3^

### 

Data collection: *APEX2* (Bruker, 2008 ▶); cell refinement: *SAINT* (Bruker, 2008 ▶); data reduction: *SAINT*; program(s) used to solve structure: *SHELXS97* (Sheldrick, 2008 ▶); program(s) used to refine structure: *SHELXL97* (Sheldrick, 2008 ▶); molecular graphics: *ORTEP-3 for Windows* (Farrugia, 1997 ▶); software used to prepare material for publication: *SHELXL97*.

## Supplementary Material

Crystal structure: contains datablocks global, I. DOI: 10.1107/S1600536811008804/ez2228sup1.cif

Structure factors: contains datablocks I. DOI: 10.1107/S1600536811008804/ez2228Isup2.hkl

Additional supplementary materials:  crystallographic information; 3D view; checkCIF report

## Figures and Tables

**Table 1:** Hydrogen-bond geometry (Å, °)

*D*—H⋯*A*	*D*—H	H⋯*A*	*D*⋯*A*	*D*—H⋯*A*
C12—H12⋯O2	0.98	2.56	3.486 (7)	157
C2—H2⋯O6^i^	0.98	2.58	3.505 (7)	158
C2—H2⋯Br1^ii^	0.98	3.06	3.718 (6)	126

Symmetry codes: (i) 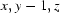; (ii) 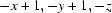.

**Fig. 1.**
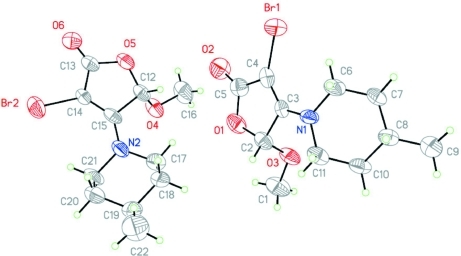
The molecular structure of the title compound showing the atom-labelling scheme. Ellipsoids are drawn at the 50% probability level.

**Fig. 2.**
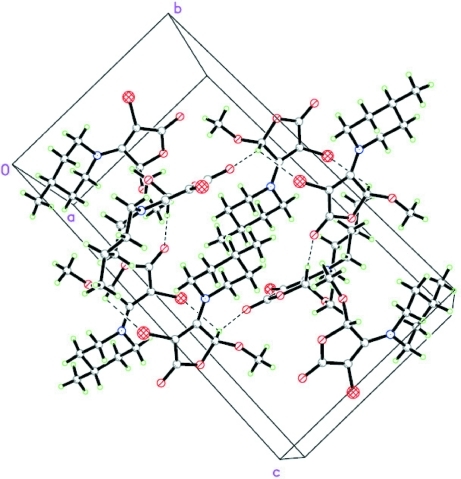
Perspective view of the crystal packing. Dashed lines correspond to hydrogen bonds.

## supplementary crystallographic information

### Comment

2(5H)-Furanone structures are found in many natural and synthetic products (Zhou *et al.*, 2009). At the same time, 4-amino-2(5H)-furanones have shown antibacterial activity and antibiotic activity against MRSA (Lattmann *et al.*, 1999; Lattmann *et al.*, 2006; Lattmann *et al.*, 2005).

Attracted by versatile 4-amino-2(5H)-furanones, we synthesized the title compound with 3,4-dibromo-5-methoxyfuran-2(5H)-one and 4-methylpiperidine in the presence of potassium fluoride *via* the tandem Michael addition-elimination reaction. Due to the presence of the 2(5H)-furanone moiety and polyfunctional groups (carboxyl, amino, halo) the title compound is expected to be a biologically active product and excellent ligand.

In the title compound, (I), two crystallographically independent molecules with R and S spatial configurations are present in the asymmetric unit. The furanone ring is approximately planar, similar to that found in a related compound (Song, Li *et al.* 2009). Additionally, the molecules are linked by weak C—H···O and C—H···Br intermolecular interactions.

### Experimental

The precursor 3,4-dibromo-5-methoxyfuran-2(5H)-furanone was prepared according to the literature procedure (Song, Wang *et al.*, 2009). After the mixture of 3,4-dibromo-5-methoxyfuran-2(5H)-furanone (2.0 mmol) and potassium fluoride (6.0 mmol) was dissolved in absolute tetrahydrofuran (2.0 mL) under nitrogen atmosphere, a tetrahydrofuran solution of 4-methylpiperidine (2.0 mmol) was added. The reaction was carried out under stirring at room temperature for 48 h. Once the reaction was complete, the solvents were removed under reduced pressure. The residual solid was dissolved in dichloromethane and extracted. The combined organic layers from the extraction were concentrated under reduced pressure, and the crude product was purified by silica gel column chromatography with a gradient mixture of petroleum ether and ethyl acetate to give the product yielding (I) 0.2988 g (51.7%).

### Refinement

H atoms were positioned in calculated positions with C—H = 0.93-0.98 Å and were refined using a riding model, with U_iso_(H) = 1.5U_eq_(C) for methyl and 1.2U_eq_(C) for the others.

### Crystal data

**Table:**

C_11_H_16_BrNO_3_	*F*(000) = 1184.0
*M**_r_* = 290.15	*D*_x_ = 1.494 Mg m^−^^3^
Monoclinic, *P*2_1_/*c*	Mo *K*α radiation, λ = 0.71073 Å
Hall symbol: -P 2ybc	Cell parameters from 1634 reflections
*a* = 12.681 (3) Å	θ = 2.4–19.9°
*b* = 10.481 (2) Å	µ = 3.18 mm^−^^1^
*c* = 19.947 (4) Å	*T* = 298 K
β = 103.312 (3)°	Block, colourless
*V* = 2579.9 (9) Å^3^	0.32 × 0.22 × 0.20 mm
*Z* = 8	

### Data collection

**Table:**

Bruker APEXII area-detector diffractometer	4532 independent reflections
Radiation source: fine-focus sealed tube	1918 reflections with *I* > 2σ(*I*)
graphite	*R*_int_ = 0.071
φ and ω scans	θ_max_ = 25.0°, θ_min_ = 1.7°
Absorption correction: multi-scan (*SADABS*; Sheldrick, 1996)	*h* = −12→15
*T*_min_ = 0.436, *T*_max_ = 0.529	*k* = −12→10
9692 measured reflections	*l* = −21→23

### Refinement

**Table:**

Refinement on *F*^2^	Primary atom site location: structure-invariant direct methods
Least-squares matrix: full	Secondary atom site location: difference Fourier map
*R*[*F*^2^ > 2σ(*F*^2^)] = 0.049	Hydrogen site location: inferred from neighbouring sites
*wR*(*F*^2^) = 0.109	H-atom parameters constrained
*S* = 1.08	*w* = 1/[σ^2^(*F*_o_^2^) + (0.010*P*)^2^] where *P* = (*F*_o_^2^ + 2*F*_c_^2^)/3
4532 reflections	(Δ/σ)_max_ = 0.002
294 parameters	Δρ_max_ = 0.53 e Å^−^^3^
0 restraints	Δρ_min_ = −0.40 e Å^−^^3^

### Special details

**Table:**

Experimental. Data for (I): ^1^H NMR (400 MHz, CDCl_3_, TMS): 0.988 (3H, *d*, *J* = 6.4 Hz, CH_3_), 1.194-1.781 (5H, *m*, CH, 2CH_2_), 2.968-3.078 (2H, *m*, CH_2_), 3.488 (3H, *s*, CH_3_), 4.301-4.362 (2H, *m*, CH_2_), 5.703(1H, *s*, CH), ESI-MS, *m*/*z* (%): Calcd for C_11_H_16_BrNO_3_^+^([M+H]^+^): 290.03(100.0), 292.03(97.0), found: 290.38(62.0), 290.32(61.5).
Geometry. All esds (except the esd in the dihedral angle between two l.s. planes) are estimated using the full covariance matrix. The cell esds are taken into account individually in the estimation of esds in distances, angles and torsion angles; correlations between esds in cell parameters are only used when they are defined by crystal symmetry. An approximate (isotropic) treatment of cell esds is used for estimating esds involving l.s. planes.
Refinement. Refinement of F^2^ against ALL reflections. The weighted R-factor wR and goodness of fit S are based on F^2^, conventional R-factors R are based on F, with F set to zero for negative F^2^. The threshold expression of F^2^ > 2sigma(F^2^) is used only for calculating R-factors(gt) etc. and is not relevant to the choice of reflections for refinement. R-factors based on F^2^ are statistically about twice as large as those based on F, and R- factors based on ALL data will be even larger.

### Fractional atomic coordinates and isotropic or equivalent isotropic displacement parameters (Å^2^)

**Table:**

	*x*	*y*	*z*	*U*_iso_*/*U*_eq_	
Br2	1.00048 (5)	1.05226 (7)	0.10092 (4)	0.1027 (3)	
Br1	0.31941 (5)	0.60499 (6)	0.02155 (3)	0.0912 (3)	
O1	0.6037 (3)	0.5375 (5)	0.1551 (2)	0.0872 (12)	
O3	0.5606 (3)	0.3404 (4)	0.1959 (2)	0.0843 (12)	
O2	0.5209 (4)	0.7230 (5)	0.1260 (2)	0.1044 (15)	
C5	0.5192 (6)	0.6083 (8)	0.1194 (3)	0.0754 (18)	
C3	0.4753 (4)	0.4075 (7)	0.0838 (3)	0.0568 (15)	
C4	0.4409 (5)	0.5293 (7)	0.0769 (3)	0.0659 (16)	
C2	0.5806 (4)	0.4055 (7)	0.1396 (3)	0.0721 (17)	
H2	0.6391	0.3649	0.1227	0.087*	
C1	0.6544 (5)	0.3198 (6)	0.2495 (3)	0.110 (2)	
H1A	0.6972	0.2529	0.2363	0.165*	
H1B	0.6330	0.2958	0.2908	0.165*	
H1C	0.6963	0.3969	0.2574	0.165*	
N1	0.4366 (3)	0.2963 (5)	0.0557 (2)	0.0683 (13)	
C7	0.2661 (4)	0.1774 (6)	0.0317 (3)	0.0731 (17)	
H7A	0.1984	0.1664	−0.0022	0.088*	
H7B	0.2488	0.2032	0.0746	0.088*	
C6	0.3293 (4)	0.2803 (6)	0.0083 (3)	0.0827 (18)	
H6A	0.3388	0.2601	−0.0374	0.099*	
H6B	0.2894	0.3598	0.0055	0.099*	
C9	0.2606 (5)	−0.0513 (6)	0.0690 (3)	0.103 (2)	
H9A	0.2400	−0.0211	0.1096	0.155*	
H9B	0.3043	−0.1265	0.0800	0.155*	
H9C	0.1967	−0.0712	0.0342	0.155*	
C10	0.4353 (4)	0.0719 (6)	0.0911 (3)	0.0815 (19)	
H10A	0.4256	0.0943	0.1365	0.098*	
H10B	0.4767	−0.0066	0.0952	0.098*	
C8	0.3248 (4)	0.0513 (6)	0.0424 (3)	0.0691 (16)	
H8	0.3367	0.0231	−0.0021	0.083*	
C11	0.4969 (4)	0.1773 (6)	0.0647 (3)	0.0811 (18)	
H11A	0.5659	0.1904	0.0970	0.097*	
H11B	0.5113	0.1517	0.0209	0.097*	
O5	0.7183 (3)	1.0359 (4)	0.1600 (2)	0.0824 (12)	
O4	0.7657 (3)	0.8990 (4)	0.25277 (19)	0.0886 (12)	
O6	0.7896 (4)	1.2032 (4)	0.1166 (2)	0.1009 (16)	
C14	0.8835 (4)	1.0052 (7)	0.1380 (3)	0.0617 (16)	
C12	0.7516 (4)	0.9100 (6)	0.1824 (3)	0.0692 (16)	
H12	0.6996	0.8466	0.1583	0.083*	
C16	0.6662 (5)	0.8945 (7)	0.2747 (3)	0.124 (2)	
H16A	0.6295	0.9749	0.2652	0.186*	
H16B	0.6811	0.8776	0.3233	0.186*	
H16C	0.6212	0.8280	0.2503	0.186*	
C13	0.7992 (5)	1.0965 (8)	0.1355 (3)	0.0693 (18)	
C15	0.8619 (4)	0.8930 (8)	0.1651 (3)	0.0644 (16)	
N2	0.9149 (4)	0.7825 (6)	0.1810 (3)	0.0830 (15)	
C18	0.8795 (5)	0.5583 (6)	0.1524 (4)	0.095 (2)	
H18A	0.8384	0.5771	0.1061	0.114*	
H18B	0.8503	0.4808	0.1676	0.114*	
C19	0.9979 (5)	0.5364 (7)	0.1510 (3)	0.092 (2)	
H19	1.0364	0.5084	0.1970	0.110*	
C17	0.8655 (5)	0.6668 (7)	0.1997 (3)	0.099 (2)	
H17A	0.7889	0.6814	0.1964	0.118*	
H17B	0.8989	0.6443	0.2470	0.118*	
C21	1.0304 (4)	0.7653 (6)	0.1837 (4)	0.107 (2)	
H21A	1.0684	0.7449	0.2305	0.129*	
H21B	1.0602	0.8442	0.1705	0.129*	
C20	1.0469 (5)	0.6595 (7)	0.1359 (4)	0.102 (2)	
H20A	1.0150	0.6843	0.0888	0.122*	
H20B	1.1240	0.6473	0.1401	0.122*	
C22	1.0082 (6)	0.4312 (7)	0.0999 (4)	0.153 (3)	
H22A	0.9635	0.4515	0.0554	0.230*	
H22B	0.9853	0.3515	0.1155	0.230*	
H22C	1.0824	0.4246	0.0966	0.230*	

### Atomic displacement parameters (Å^2^)

**Table:**

	*U*^11^	*U*^22^	*U*^33^	*U*^12^	*U*^13^	*U*^23^
Br2	0.0775 (5)	0.1390 (7)	0.1017 (6)	−0.0027 (4)	0.0413 (4)	−0.0018 (4)
Br1	0.0840 (5)	0.0931 (5)	0.0904 (5)	0.0209 (4)	0.0077 (4)	0.0021 (4)
O1	0.062 (3)	0.103 (4)	0.088 (3)	−0.009 (3)	0.001 (2)	−0.025 (3)
O3	0.054 (2)	0.132 (4)	0.062 (3)	0.015 (2)	0.003 (2)	0.006 (2)
O2	0.104 (4)	0.088 (4)	0.117 (4)	−0.008 (3)	0.017 (3)	−0.025 (3)
C5	0.076 (5)	0.083 (5)	0.076 (5)	0.010 (5)	0.034 (4)	−0.009 (5)
C3	0.049 (4)	0.063 (5)	0.060 (4)	−0.013 (4)	0.013 (3)	−0.008 (3)
C4	0.065 (4)	0.084 (5)	0.052 (4)	−0.023 (4)	0.021 (3)	−0.016 (4)
C2	0.055 (4)	0.105 (6)	0.054 (4)	0.003 (4)	0.009 (3)	−0.003 (4)
C1	0.085 (5)	0.176 (7)	0.055 (4)	0.022 (4)	−0.011 (4)	0.004 (4)
N1	0.051 (3)	0.065 (4)	0.077 (3)	0.010 (3)	−0.010 (3)	0.004 (3)
C7	0.050 (4)	0.094 (5)	0.064 (4)	0.002 (4)	−0.009 (3)	−0.005 (3)
C6	0.068 (4)	0.089 (5)	0.070 (4)	0.003 (4)	−0.028 (3)	−0.003 (3)
C9	0.082 (5)	0.106 (5)	0.117 (6)	0.004 (4)	0.013 (4)	0.002 (4)
C10	0.063 (4)	0.088 (5)	0.080 (4)	0.023 (4)	−0.009 (4)	0.000 (3)
C8	0.062 (4)	0.073 (4)	0.068 (4)	0.014 (4)	0.006 (3)	−0.011 (3)
C11	0.058 (4)	0.097 (5)	0.082 (4)	0.011 (4)	0.002 (3)	−0.019 (4)
O5	0.050 (3)	0.108 (4)	0.087 (3)	0.009 (3)	0.010 (2)	0.002 (3)
O4	0.061 (3)	0.156 (4)	0.049 (3)	−0.007 (2)	0.013 (2)	−0.003 (2)
O6	0.088 (3)	0.092 (4)	0.123 (4)	0.018 (3)	0.027 (3)	0.011 (3)
C14	0.045 (4)	0.084 (5)	0.056 (4)	−0.001 (4)	0.011 (3)	−0.005 (3)
C12	0.047 (4)	0.111 (5)	0.050 (4)	0.002 (4)	0.011 (3)	−0.014 (4)
C16	0.101 (6)	0.197 (7)	0.090 (5)	−0.003 (5)	0.056 (4)	0.010 (5)
C13	0.052 (4)	0.093 (6)	0.062 (4)	0.012 (5)	0.011 (3)	−0.014 (4)
C15	0.040 (4)	0.099 (5)	0.049 (3)	0.008 (4)	−0.002 (3)	−0.021 (4)
N2	0.044 (3)	0.090 (4)	0.112 (4)	0.000 (3)	0.010 (3)	−0.002 (3)
C18	0.064 (5)	0.089 (5)	0.122 (6)	0.007 (4)	−0.001 (4)	0.024 (4)
C19	0.076 (5)	0.083 (5)	0.105 (5)	0.011 (4)	−0.003 (4)	0.018 (4)
C17	0.077 (5)	0.112 (6)	0.103 (6)	0.013 (5)	0.013 (4)	0.025 (5)
C21	0.037 (4)	0.101 (6)	0.170 (7)	0.003 (4)	−0.007 (4)	−0.004 (5)
C20	0.067 (5)	0.095 (6)	0.142 (6)	0.018 (5)	0.024 (4)	0.005 (5)
C22	0.160 (9)	0.116 (7)	0.195 (9)	0.006 (5)	0.065 (7)	−0.042 (6)

### Geometric parameters (Å, °)

**Table:**

Br2—C14	1.870 (6)	C11—H11B	0.9700
Br1—C4	1.855 (6)	O5—C13	1.388 (7)
O1—C5	1.362 (7)	O5—C12	1.425 (6)
O1—C2	1.433 (6)	O4—C12	1.379 (6)
O3—C2	1.387 (6)	O4—C16	1.428 (6)
O3—C1	1.420 (6)	O6—C13	1.178 (6)
O2—C5	1.209 (6)	C14—C15	1.348 (7)
C5—C4	1.414 (8)	C14—C13	1.427 (8)
C3—N1	1.337 (6)	C12—C15	1.526 (7)
C3—C4	1.345 (7)	C12—H12	0.9800
C3—C2	1.529 (7)	C16—H16A	0.9600
C2—H2	0.9800	C16—H16B	0.9600
C1—H1A	0.9600	C16—H16C	0.9600
C1—H1B	0.9600	C15—N2	1.340 (7)
C1—H1C	0.9600	N2—C17	1.452 (7)
N1—C11	1.452 (6)	N2—C21	1.464 (6)
N1—C6	1.476 (6)	C18—C17	1.514 (7)
C7—C6	1.481 (7)	C18—C19	1.526 (8)
C7—C8	1.508 (6)	C18—H18A	0.9700
C7—H7A	0.9700	C18—H18B	0.9700
C7—H7B	0.9700	C19—C20	1.492 (8)
C6—H6A	0.9700	C19—C22	1.527 (8)
C6—H6B	0.9700	C19—H19	0.9800
C9—C8	1.517 (7)	C17—H17A	0.9700
C9—H9A	0.9600	C17—H17B	0.9700
C9—H9B	0.9600	C21—C20	1.509 (8)
C9—H9C	0.9600	C21—H21A	0.9700
C10—C11	1.517 (7)	C21—H21B	0.9700
C10—C8	1.525 (7)	C20—H20A	0.9700
C10—H10A	0.9700	C20—H20B	0.9700
C10—H10B	0.9700	C22—H22A	0.9600
C8—H8	0.9800	C22—H22B	0.9600
C11—H11A	0.9700	C22—H22C	0.9600
			
C5—O1—C2	108.4 (5)	C13—O5—C12	110.1 (5)
C2—O3—C1	114.0 (4)	C12—O4—C16	113.4 (4)
O2—C5—O1	119.5 (7)	C15—C14—C13	112.3 (6)
O2—C5—C4	129.7 (7)	C15—C14—Br2	129.9 (5)
O1—C5—C4	110.7 (6)	C13—C14—Br2	117.7 (5)
N1—C3—C4	134.9 (5)	O4—C12—O5	110.8 (5)
N1—C3—C2	117.9 (6)	O4—C12—C15	108.0 (4)
C4—C3—C2	107.2 (5)	O5—C12—C15	105.0 (5)
C3—C4—C5	109.1 (6)	O4—C12—H12	111.0
C3—C4—Br1	132.2 (4)	O5—C12—H12	111.0
C5—C4—Br1	118.6 (6)	C15—C12—H12	111.0
O3—C2—O1	111.5 (5)	O4—C16—H16A	109.5
O3—C2—C3	107.7 (5)	O4—C16—H16B	109.5
O1—C2—C3	104.1 (5)	H16A—C16—H16B	109.5
O3—C2—H2	111.1	O4—C16—H16C	109.5
O1—C2—H2	111.1	H16A—C16—H16C	109.5
C3—C2—H2	111.1	H16B—C16—H16C	109.5
O3—C1—H1A	109.5	O6—C13—O5	121.1 (6)
O3—C1—H1B	109.5	O6—C13—C14	132.1 (7)
H1A—C1—H1B	109.5	O5—C13—C14	106.8 (6)
O3—C1—H1C	109.5	N2—C15—C14	135.3 (6)
H1A—C1—H1C	109.5	N2—C15—C12	119.0 (6)
H1B—C1—H1C	109.5	C14—C15—C12	105.7 (6)
C3—N1—C11	124.4 (5)	C15—N2—C17	124.0 (5)
C3—N1—C6	124.2 (5)	C15—N2—C21	123.9 (6)
C11—N1—C6	111.2 (4)	C17—N2—C21	112.0 (5)
C6—C7—C8	113.4 (5)	C17—C18—C19	112.4 (5)
C6—C7—H7A	108.9	C17—C18—H18A	109.1
C8—C7—H7A	108.9	C19—C18—H18A	109.1
C6—C7—H7B	108.9	C17—C18—H18B	109.1
C8—C7—H7B	108.9	C19—C18—H18B	109.1
H7A—C7—H7B	107.7	H18A—C18—H18B	107.8
N1—C6—C7	111.6 (4)	C20—C19—C18	109.3 (5)
N1—C6—H6A	109.3	C20—C19—C22	112.7 (7)
C7—C6—H6A	109.3	C18—C19—C22	110.9 (6)
N1—C6—H6B	109.3	C20—C19—H19	107.9
C7—C6—H6B	109.3	C18—C19—H19	107.9
H6A—C6—H6B	108.0	C22—C19—H19	107.9
C8—C9—H9A	109.5	N2—C17—C18	110.3 (6)
C8—C9—H9B	109.5	N2—C17—H17A	109.6
H9A—C9—H9B	109.5	C18—C17—H17A	109.6
C8—C9—H9C	109.5	N2—C17—H17B	109.6
H9A—C9—H9C	109.5	C18—C17—H17B	109.6
H9B—C9—H9C	109.5	H17A—C17—H17B	108.1
C11—C10—C8	110.6 (5)	N2—C21—C20	110.5 (5)
C11—C10—H10A	109.5	N2—C21—H21A	109.5
C8—C10—H10A	109.5	C20—C21—H21A	109.5
C11—C10—H10B	109.5	N2—C21—H21B	109.5
C8—C10—H10B	109.5	C20—C21—H21B	109.5
H10A—C10—H10B	108.1	H21A—C21—H21B	108.1
C7—C8—C9	112.6 (5)	C19—C20—C21	112.5 (6)
C7—C8—C10	108.5 (4)	C19—C20—H20A	109.1
C9—C8—C10	111.5 (5)	C21—C20—H20A	109.1
C7—C8—H8	108.0	C19—C20—H20B	109.1
C9—C8—H8	108.0	C21—C20—H20B	109.1
C10—C8—H8	108.0	H20A—C20—H20B	107.8
N1—C11—C10	111.7 (5)	C19—C22—H22A	109.5
N1—C11—H11A	109.3	C19—C22—H22B	109.5
C10—C11—H11A	109.3	H22A—C22—H22B	109.5
N1—C11—H11B	109.3	C19—C22—H22C	109.5
C10—C11—H11B	109.3	H22A—C22—H22C	109.5
H11A—C11—H11B	107.9	H22B—C22—H22C	109.5
			
C2—O1—C5—O2	178.6 (5)	C16—O4—C12—O5	74.2 (6)
C2—O1—C5—C4	−3.1 (6)	C16—O4—C12—C15	−171.3 (5)
N1—C3—C4—C5	−178.2 (6)	C13—O5—C12—O4	112.8 (5)
C2—C3—C4—C5	4.6 (6)	C13—O5—C12—C15	−3.5 (5)
N1—C3—C4—Br1	−1.2 (10)	C12—O5—C13—O6	−177.4 (5)
C2—C3—C4—Br1	−178.5 (4)	C12—O5—C13—C14	3.7 (5)
O2—C5—C4—C3	177.0 (6)	C15—C14—C13—O6	178.8 (6)
O1—C5—C4—C3	−1.1 (6)	Br2—C14—C13—O6	−4.0 (9)
O2—C5—C4—Br1	−0.5 (9)	C15—C14—C13—O5	−2.5 (6)
O1—C5—C4—Br1	−178.5 (4)	Br2—C14—C13—O5	174.7 (3)
C1—O3—C2—O1	−72.3 (6)	C13—C14—C15—N2	−176.5 (5)
C1—O3—C2—C3	174.1 (5)	Br2—C14—C15—N2	6.8 (9)
C5—O1—C2—O3	−110.3 (5)	C13—C14—C15—C12	0.3 (6)
C5—O1—C2—C3	5.5 (6)	Br2—C14—C15—C12	−176.4 (4)
N1—C3—C2—O3	−65.6 (6)	O4—C12—C15—N2	61.0 (6)
C4—C3—C2—O3	112.3 (5)	O5—C12—C15—N2	179.3 (4)
N1—C3—C2—O1	176.0 (4)	O4—C12—C15—C14	−116.4 (5)
C4—C3—C2—O1	−6.2 (6)	O5—C12—C15—C14	1.9 (5)
C4—C3—N1—C11	170.9 (6)	C14—C15—N2—C17	−169.3 (6)
C2—C3—N1—C11	−12.0 (8)	C12—C15—N2—C17	14.2 (7)
C4—C3—N1—C6	−5.5 (9)	C14—C15—N2—C21	12.8 (10)
C2—C3—N1—C6	171.6 (5)	C12—C15—N2—C21	−163.7 (5)
C3—N1—C6—C7	−127.5 (5)	C17—C18—C19—C20	−52.1 (7)
C11—N1—C6—C7	55.6 (6)	C17—C18—C19—C22	−177.0 (6)
C8—C7—C6—N1	−55.0 (6)	C15—N2—C17—C18	123.8 (6)
C6—C7—C8—C9	177.7 (5)	C21—N2—C17—C18	−58.1 (6)
C6—C7—C8—C10	53.9 (6)	C19—C18—C17—N2	55.1 (7)
C11—C10—C8—C7	−53.9 (6)	C15—N2—C21—C20	−123.3 (6)
C11—C10—C8—C9	−178.4 (5)	C17—N2—C21—C20	58.7 (7)
C3—N1—C11—C10	125.8 (5)	C18—C19—C20—C21	52.5 (7)
C6—N1—C11—C10	−57.4 (6)	C22—C19—C20—C21	176.4 (6)
C8—C10—C11—N1	57.5 (6)	N2—C21—C20—C19	−56.3 (7)

### Hydrogen-bond geometry (Å, °)

**Table:**

*D*—H···*A*	*D*—H	H···*A*	*D*···*A*	*D*—H···*A*
C12—H12···O2	0.98	2.56	3.486 (7)	157
C2—H2···O6^i^	0.98	2.58	3.505 (7)	158
C2—H2···Br1^ii^	0.98	3.06	3.718 (6)	126

Symmetry codes: (i) *x*, *y*−1, *z*; (ii) −*x*+1, −*y*+1, −*z*.
